# Supplementary material for: Best Practices for Notification of Unexpected, Violent, and Traumatic Death: A Scoping Review
Source: Int J Environ Res Public Health. 2023 Jun 25;20(13):6222. doi: 10.3390/ijerph20136222 (PMC10341669; doi:10.3390/ijerph20136222)
Supplement: Supplementary file 1 [file ijerph-20-06222-s001.zip › ijerph-2208349-supplementary.pdf]

**Supplemental material**

Below is the complete bibliographic search strategy, including all the filters used, for one of the databases that provided the most consistent data from the search: Ebsco Psychinfo.

- Access to the Ebsco portal was made through the Proxy of the University of Padua;
- The database was selected: PsycInfo;
- The keyword 01 is entered in quotation marks in the first search bar;
- Each author limited the research to the range of years assigned to them; for example, Zammarrelli initially selected 1991 – 1993; then she repeated the search for the period 2003 – 2005, and finally she repeated the search for the period 2015 – 2017;
- No limit has been selected with respect to the options: Linked Full Text; References Available; Open Access;
- In the type of resource, the option is selected: all results;
- The records found were copied and pasted into a word file;
- Enter the keyword 02 in quotation marks in the first search bar;
- The same research methodology is repeated;
- The records found were copied and pasted into a word file;
- The keyword 03 is entered in quotation marks in the first search bar;
- The same research methodology is repeated;
- The records found were copied and pasted into a word file;
- The keyword 04 is entered in quotation marks in the first search bar;
- The same research methodology is repeated;
- The records found were copied and pasted into a word file;
- For the keyword 05, the first term (notification) is entered in the first search bar;
- The first AND option is used by entering "traumatic death" in the search bar in quotation marks;
- The same research methodology is repeated;
- The records found were copied and pasted into a word file;
- For the keyword 06, the first term (communication) is entered in the first search bar;
- The first AND option is used by entering "traumatic death" in the search bar in quotation marks;
- The same research methodology is repeated;
- The records found were copied and pasted into a word file;
- For the keyword 07 the first term (notification) is entered in the first search bar;
- The first AND option is used by entering “sudden death” in the search bar in quotation marks;
- The same research methodology is repeated;
- The records found were copied and pasted into a word file;
- For the keyword 08 the first term (communication) is entered in the first search bar;

- The first AND option is used by entering “sudden death” in the search bar in quotation marks;
- The same research methodology is repeated;
- The records found were copied and pasted into a word file. (item 8)
